# A Handbook of the Diseases of Children and Their Homœopathic Treatment

**Published:** 1896-02

**Authors:** 


A Handbook of the Diseases of Children and their Homœopathic Treatment. Illustrated. A text-book for students, colleges, and physicians. By Charles E. Fisher, M. D., President of The American Institute of Homœopathy, and Editor of *The Medical Century*. Chicago: Medical Century Co., 31 Washington Street. 1895. Price, cloth, $5.00; leather, $6.00; half morocco, $7.00.

This elegant volume of nine hundred pages is probably the best and most complete work on the diseases of childhood that has been given to the homœopathic school.

The author says in his preface that the motive for his writing the book was a conviction "that there exists a vacancy in the literature of Homœopathy in the department of pediatrics which demands to be filled."

He further says: "Written from the view-point of the bedside practitioner, the book is intended to serve as a text-book for students and beginners in practice and a work of ready reference and resourceful comfort for the busy doctor of the general field as well. More than twenty years of the author's professional life have been spent in sections where homœopathic physicians were few and their system comparatively unknown, with consultants not often at hand, self-reliance being demanded. For those similarly situated and also for those who prefer to work out their own salvation in difficult cases the book has been prepared. It is believed it is more complete than any similar volume of its profession, though, doubtless, lacking in many respects and far from perfect."

The reviewer can cordially add that his own conviction agrees with that of the distinguished author.

Looking through the volume, we observe that the author is not greatly in favor of artificial foods for children, though he does say which he prefers among them. He also denounces the professional wet nurse. On the other hand, he thinks very well of the milk of the jennet for young children. He also suggests the milk of the sheep for children. Both of these milks he regarded as preferable to cow's milk, in the absence of the mother's own milk. His reasons are clearly stated and of course are perfectly sound.

He recommends that the new-born child be not washed with water for the first two or three days, but anointed with sweet oil instead. His reasons for this procedure are the danger of broncho-pneumonia, or of fatal congestion of internal organs from the removal of the protective oil of the skin.

The directions for treatment of asphyxia in the new born are clearly stated, and illustrated with drawings. He has no use for the flannel belly-band of children, which he considers as tending to prevent normal distention of the abdomen arising from the formation of gases, and so being one of the prolific causes of infantile colic. That it is liable to adhere to the navel before the separation of the fragment of the cord, and being thus accidentally pulled upon causes discomfort to the child, and favors the occurrence of umbilical hernia.

In ophthalmia his main reliance for treatment is upon germicides, the indications for homœopathic remedies being rather scant.

In the description of scarlet fever, the author advocates the "Tonsillar Theory." According to this theory "scarlet fever is due to the invasion of the crypts of the tonsils by the *streptococcus pyogenes*, or common pus-producing bacillus, no matter what its origin, and the consequent development of a strictly septic fever of varying intensity and malignancy." In support of this theory the fact is cited that "children with enlarged tonsils are especially liable to the disease; that with the atrophy of these glands at the age of puberty, liability to attack by scarlet fever disappears; and that even malignant cases in large families may not result in the infection of other children."

Excellent descriptions of the fever follow, and the question of prophylaxis is discussed. Indications for homœopathic remedies are freely given, and such adjuvants as oxygen gas, when in the state of coma and putrescence, are advised. Cold bathing is repudiated as being absolutely dangerous, and a diet of hot milk, or hot water and cream recommended. Bovinine as an enema is also proposed along with milk. Meat-broth, beef-tea, and chicken-broth are also directed, and finally the possibility of an aggravation of nephritic tendency by the use of too much of beef and mutton broths is noticed.

In diphtheria the author shows a friendliness to Antitoxin, which seems to us out of place in view of its dangers and failures, and the successes achieved by the prescription of the similar remedy.

He also advocates vaccination in small-pox, and repeats a lot of statistics highly favorable to the practice of it. He attributes the failures to stave off small-pox in a great many cases that have been previously vaccinated, to the fact that in such cases the vaccination had been performed only once, or if oftener, not within six or seven years of the attack of small-pox. He thinks the best evidence of the value of vaccination lies in the fact that before the time of Jenner, small-pox was considered particularly the disease of childhood, like measles and scarlet fever; and now that vaccination has become universal, it is no longer so regarded. He winds up with the announcement that in the absence of more convincing information to the contrary, he is in favor of vaccination.

These points are perhaps among the most striking in the book. For the rest, all the usual diseases of childhood are noticed, copiously illustrated, and the homœopathic indications also given. These last are not sufficiently free for the advanced practitioner, and so he must of course go to the fountain-head, the *Materia Medica Pura*. The indications given are largely made up from Dr. Guernsey's *Keynotes*, which have been so justly prized by a large part of the profession. Altogether, Dr. Fisher's book is a great success, and should be cordially received by the profession.

